# Testing the Link between Functional Diversity and Ecosystem Functioning in a Minnesota Grassland Experiment

**DOI:** 10.1371/journal.pone.0052821

**Published:** 2012-12-31

**Authors:** Christopher M. Clark, Dan F. B. Flynn, Bradley J. Butterfield, Peter B. Reich

**Affiliations:** 1 School of Life Sciences and Global Institute of Sustainability, Arizona State University, Tempe, Arizona, United States of America; 2 Department of Ecology, Evolution, and Environmental Biology, Columbia University, New York, New York, United States of America; 3 Merriam-Powell Center for Environmental Research and Department of Biology, Northern Arizona University, Flagstaff, Arizona, United States of America; 4 Department of Forest Resources, University of Minnesota, St. Paul, Minnesota, United States of America; University of Alberta, Canada

## Abstract

The functional diversity of a community can influence ecosystem functioning and reflects assembly processes. The large number of disparate metrics used to quantify functional diversity reflects the range of attributes underlying this concept, generally summarized as functional richness, functional evenness, and functional divergence. However, in practice, we know very little about which attributes drive which ecosystem functions, due to a lack of field-based tests. Here we test the association between eight leading functional diversity metrics (Rao’s Q, FD, FDis, FEve, FDiv, convex hull volume, and species and functional group richness) that emphasize different attributes of functional diversity, plus 11 extensions of these existing metrics that incorporate heterogeneous species abundances and trait variation. We assess the relationships among these metrics and compare their performances for predicting three key ecosystem functions (above- and belowground biomass and light capture) within a long-term grassland biodiversity experiment. Many metrics were highly correlated, although unique information was captured in FEve, FDiv, and dendrogram-based measures (FD) that were adjusted by abundance. FD adjusted by abundance outperformed all other metrics in predicting both above- and belowground biomass, although several others also performed well (e.g. Rao’s Q, FDis, FDiv). More generally, trait-based richness metrics and hybrid metrics incorporating multiple diversity attributes outperformed evenness metrics and single-attribute metrics, results that were not changed when combinations of metrics were explored. For light capture, species richness alone was the best predictor, suggesting that traits for canopy architecture would be necessary to improve predictions. Our study provides a comprehensive test linking different attributes of functional diversity with ecosystem function for a grassland system.

## Introduction

Functional diversity, commonly referred to as the value, range, and distribution of functional traits of organisms in a community [1], [2], is hypothesized to reflect many processes in community and ecosystem ecology. Researchers have examined how different community assembly processes (e.g. limiting similarity, habitat filtering, neutrality) influence functional diversity [1], [3]–[6], as well as how varying levels of functional diversity influence ecosystem processes and properties [7]–[9]. Because functional diversity plays such a central role in many areas of ecological research, understanding and quantifying this concept is considered vital to a wide spectrum of research topics in ecology.

Historically, biodiversity research on plant communities has focused on the number of species within a community (species richness) as an implicit reflection of functional diversity and as a driver of ecosystem processes [8], [10]. Although increased species richness is typically associated with greater levels of ecosystem functioning [11], [12], this approach does not explicitly incorporate the traits responsible for these processes. Research over the past decade has considerably advanced the field, with at least 10 trait-based functional diversity metrics being proposed thus far (reviewed in [13]–[15]). These include the unadjusted sum (Functional Attribute Diversity, FAD; [16]) or average [17] of pair-wise distances between species in trait-space (functional dissimilarity), the abundance-weighted variance in traits using multiple traits (Rao’s quadratic entropy, *Q*; [18], [19]), the abundance-weighted variance of traits using a single trait (*FD_var_*; [20]), the regularity of trait distribution (Functional Regularity Index, *FRO*; [21]), the sum of branch lengths following cluster analysis of traits in a community (*FD*,; [22]), the volume of trait space occupied (Convex Hull Volume, *Hull*; [23]), the evenness of the abundance distribution in the minimum spanning tree linking all species (*FEve*, [24]), the divergence of abundance distributions relative to the community centroid (*FDiv*, [24]), as well as the mean distance of species from the community centroid after adjusting for abundances (*FDis*, [25]).

Unfortunately, there is no consensus at to which functional diversity measure performs best. Mason et al (2005) and Villéger et al (2008), instead, emphasize that there may not be a single “best” metric for measuring functional diversity - each has its own merits and accentuates different attributes of the concept. The question then becomes, and the one which we focus on in this study, which attribute(s) of functional diversity has a stronger influence on which ecosystem processes and under which conditions [26]? Mason et al. (2005) suggested that functional diversity can be generally deconstructed into three components: functional richness, functional evenness, and functional divergence. Functional richness indices measure the amount of trait space occupied by the community. Functional evenness indices measure how regularly that space is filled. Functional divergence measures whether species are generally clustered towards the center of the community centroid, or are more dispersed towards the edges of trait-space [14], [24]. Some ecosystem processes might be affected more by the total volume of trait space occupied, and others by the packing of species within that space. For example, if a process is dominated by disparate species, such as perennial C_4_ grasses and legumes jointly affecting production in Minnesota grasslands [27], metrics that emphasize richness or divergence might better predict that function than metrics that emphasize species evenness. If a process is influenced by species more evenly, a metric that focuses on functional evenness might outperform others. A deeper understanding of these linkages would aid conservationists and decision makers to determine which sets of species and traits affect particular ecosystem services of concern.

Unfortunately, field tests based on empirical data examining which attributes of functional diversity best predict ecosystem dynamics are relatively scarce in the literature. The few field studies to date have found that some functional attributes predict some functions in certain cases but not in others [26], [28], [29]. Mouillot et al. (2011) found in an analysis of a German grassland biodiversity-ecosystem-function study that functional identity, measured as the first three axes from a trait-based PCA, and functional diversity, measured as three metrics (FDiv, FEve, and FRic), explained most of the variation in six ecosystem processes [52]. In particular, functional divergence measured as FDiv was prominent in its explanatory ability for individual functions and ecosystem multifunctionality. However, similar analyses that incorporate multiple aspects of functional diversity in real (non-simulated) communities remain rare.

Also relatively scarce from the functional diversity literature have been efforts to combine the attributes from different approaches that have strong theoretical support. In particular, the functional richness metrics FD [22] and Convex Hull Volume [23] give equal weight to species regardless of their abundance, and could be combined with approaches that incorporate abundance to generate hybrid metrics with combined attributes. These two metrics for example have each found some successes in predicting ecosystem function and community assembly [23], [29]–[32]. However, neither adjusts a species’ influence by its relative abundance, a concept that has strong theoretical support (i.e. the “mass ratio effect”; [33]). Indeed, FD does not change unless unique species are added or lost from the community; and Hulls do not change unless these new species extend the hypervolume. Rao’s Q describes both functional richness and divergence and can be a useful summary measure that can be decomposed into alpha-, beta-, and gamma-diversities [1], [34]. Whether the blurring of these attributes is desirable or not likely depends on the needs of the user and the question being addressed.

In addition to the above considerations of abundance, functional diversity metrics ignore the fact that not all traits are equally variable. This creates an implicit assumption, for example, that a 15% change in one trait (e.g. leaf N) is ecologically equivalent to a 15% change in another (e.g. seed mass). This assumption, which we term the “homogeneous variation assumption” stems from the initial normalization procedure that all metrics utilize in order to generate scale neutrality. This assumption stands somewhat at odds with the notion that functional diversity is influenced by the variation of traits in the community (which may differ for different traits), and remains untested with very few exceptions (e.g. [23]).

Thus, there are many issues that remain unresolved in terms of the linkages between functional diversity and ecosystem function in real systems. We address several of these, centered around a single experiment, in an effort to synthesize greater understanding than a piecemeal approach would afford. Here we use long-term field data from a grassland biodiversity experiment to (1) test which attributes of functional diversity more closely describe two prominent ecosystem functions (aboveground biomass and light capture), and (2) incorporate into this test hybrid metrics, or augmentations to existing metrics, that incorporate heterogeneous variation among traits and abundance-weighting to FD and Convex Hulls. It is not the goal of this effort to find the best functional diversity metric for all systems or all processes, but rather to gain more understanding of which attributes of functional diversity, embodied to different degrees by different metrics, map to these two ecosystem functions.

## Materials and Methods

### Plant Community and Trait Data

We used plant-community and species-trait data from a 10 year experiment in Minnesota designed to examine the effect of plant biodiversity and global change (elevated versus ambient CO_2_ and N) on grassland function [35], [36]. We focus on plots receiving ambient CO_2_ and N treatments for the present study. Thus, we only used data from 59 plots (2 m×2 m) which were planted with 4, 9, and 16 species under ambient conditions. The 16 species used in this study were all native or naturalized to the Cedar Creek Ecosystem Science Reserve. They include four C4 grasses (*Andropogon gerardii*, *Bouteloua gracilis*, *Schizachyrium scoparium*, *Sorghastrum nutans*), four C3 grasses (*Agropyron repens*, *Bromus inermis*, *Koeleria cristata*, *Poa pratensis*), four N-fixing legumes (*Amorpha canescens*, *Lespedeza capitata*, *Lupinus perennis*, *Petalostemum villosum*) and four non-N-fixing herbaceous species (*Achillea millefolium*, *Anemone cylindrica*, *Asclepias tuberosa*, *Solidago rigida*), and all are referred to by genus elsewhere.

A trait-based approach to predicting ecosystem function involves defining a function of interest, determining predictive traits for that function, and measuring representative values for those traits (summarized in [13]). We were most interested in functions associated with plant growth and biomass production, and focused on aboveground biomass as our primary function of interest. Additionally, we assessed the ability of functional diversity metrics to predict light interception and belowground biomass in order to test the transferability of the process between related functions. We compiled a list of candidate traits based on previous work here and elsewhere, and on availability of trait data, which included specific leaf area, leaf nitrogen concentration (by mass), specific root length, height, N-fixation ability, seed mass, and root mass fraction. Many of these traits have been found to be collinear in trait screening studies [37]–[39], with a smaller set of traits desired for predicting function [13]. For trait numbers, we were somewhat restricted by a dimensionality requirement of Hulls in that there must be more species (S) than traits (T) to define a unique Hull volume (S_min_>Num(T)). Thus, with a lowest richness treatment of 4 species, we could have no more than 3 traits for comparison across diversity metrics. To relax this restriction, we conducted additional tests excluding Hulls to incorporate a larger number of traits, as well. Specific leaf area (SLA), leaf N concentration (leaf N) and root mass fraction (RMF) capture plant strategies for resource consumption and biomass production above- and belowground, and much prior research at this and other sites have found these traits to be good predictors of functions associated with aboveground productivity [40]. These three traits were not highly correlated with one another in our dataset and were used for all subsequent calculations (range of significance values for Spearman’s ρ: 0.06–0.13). For trait values, we used data from monocultures of each species averaged over 2000 and 2001, collected using standardized protocols [41]. As an additional test, we included species mean seed mass, height, and specific root length (SRL) [42]–[44]. For aboveground biomass, plants were harvested each year in a 10×100 cm section of each plot. Clippings were sorted to live material and litter, live material was sorted to species, and all material was dried and weighed. Light was measured at peak biomass, averaging over three subsamples per plot at the soil surface relative to ambient light using an integrating light ceptometer (Decagon Devices, Inc, Pullman, WA). Additional experimental details are available in prior publications [35], [36].

### Calculation of Diversity Metrics

For each plot and each year, we calculated 8 foundational indices and 11 modified indices (Table 1). Foundational indices included Rao's quadratic entropy (Q), Villéger et al’s (2008) functional evenness and functional divergence metrics (FEve and FDiv, respectively), Laliberté and Legendre’s (2010) functional dispersion metric (FDis), Petchey and Gaston’s (2006) functional dendrogram (FD), Cornwell et al’s (2006) convex hull volume (Hull), as well as species richness (S; either assessed by planned treatment or by measured observation), and functional group richness (FGR; either assessed by planned treatment or by measured observation).

**Table 1:** Summary of diversity metrics used in this study.

Metric #	Base	Metric ID	Metric Description	Correlation: mean	Correlation: with S_trt_(#17)
1	FD	1FD	Total branch length of functional dendrogram	0.60	0.79***
2		2FD_abun_	Traits weighted by p_i_	0.17	0.03 ns
3		2FD_joint.abun_	Distance weighted by 1+p_i_p_j_	0.61	0.78***
4		2FD_cv_	Trait axes scaled by CV	0.60	0.80***
5		2FD_cv.abun_	Combination of #2 and #4	0.15	0.003 ns
6		2FD_cv.joint.abun_	Combination of #3 and #4	0.61	0.78***
7	Hull	3Hull	Minimum volume circumscribed by species in multidimensional trait-space	0.53	0.70***
8		2Hull_ abun_	Traits weighted by p_i_	0.32	0.32***
9		2Hull_cv_	Trait axes scaled by CV	0.50	0.70***
10		2Hull_ cv.abun_	Combination of #8 and #9	0.32	0.32***
11	Other	4Q	Rao's quadratic entropy	0.53	0.62***
12		2Q_cv_	CV-weighted Rao's quadratic entropy	0.53	0.62***
13		5FEve	Evenness of abundance distribution in the minimum spanning tree	−0.08	−0.16***
14		5FDiv	Divergence of abundance distributions relative to the community centroid	0.14	0.12**
15		6FDis	Mean distance of individual species to the community centroid	0.55	0.62***
16		S_obs_	Observed species richness	0.52	0.91***
17		S_trt_	Treatment species richness	0.51	
18		FGR_obs_	Observed functional group richness	0.46	0.63***
19		FRG_trt_	Treatment functional group richness	0.50	0.63***

Abundances of species *i* and *j* abbreviated *p_i_* and *p_j_*. Also shown are average correlations with the 18 other indices, and correlation with planned richness (significance denoted as: *, ns, P>0.05; *, P<0.05; **, P<0.01; ***, P<0.001).

1 Petchey OL, Gaston KJ. 2002. Functional diversity (FD), species richness and community composition. Ecology Letters 5∶402–411.

2 This study.

3 Cornwell WK, Schwilk DW, Ackerly DD. 2006. A trait-based test for habitat filtering: Convex hull volume. Ecology 87∶1465–1471.

4 Rao CR. 1982. Diversity and Dissimilarity Coefficients – A unified approach. Theoretical Population Biology 21∶24–43.

5 Villéger S, Mason NWH, Mouillot D. 2008. New multidimensional functional diversity indices for a multifaceted framework in functional ecology. Ecology 89∶2290–2301.

6 Laliberté E, Legendre P. 2010. A distance-based framework for measuring functional diversity from multiple traits. Ecology 91∶299–305.

We modified FD and Hulls each in two ways: (1) to incorporate relative abundances of the constituent species, and (2) to incorporate heterogeneous variation among traits. FD is calculated, in short, using a normalized species×trait matrix (columns are by trait and have mean zero, standard deviation unity), by calculating multivariate distances between species based on their traits, clustering those distances into a dendrogram, and summing the branch lengths in a given community [22]. This process requires several decisions, including the choice of appropriate distance metric and clustering algorithm [45], [46]. Although no single best procedure exists for all research endeavors [47], Gower’s distance is generally preferred because it can accommodate multiple data types [45]. We use Gower’s distance to enable greater generalization and future comparability of this approach. The choice of the clustering algorithm can also have consequences for the FD calculation in some cases [47]. We tested several clustering algorithms (e.g. centroid, single-linkage, Ward's minimum variance) and selected UPGMA, as it yielded a dendrogram with the highest cophenetic correlation with the original distance matrix [48]. The cophenetic correlation measures how faithfully a dendrogram preserves the original pairwise distances among multivariate data points. UPGMA has been found to often outperform other clustering algorithms (Mouchet et al. 2008). Thus, we present Gower’s distance and UPGMA clustering algorithm throughout. A Hull, in short, is calculated using a normalized species×trait matrix, as the minimum volume required to contain a set of points in trait space [23]. Thus, as originally formulated, FD does not change unless unique species are added or lost from the community. Hulls do not change unless these unique species are very different from others in the community (i.e. on the surface of the volume, species internal to the volume contribute nothing to functional diversity measured by Hulls).

**Figure 1:**
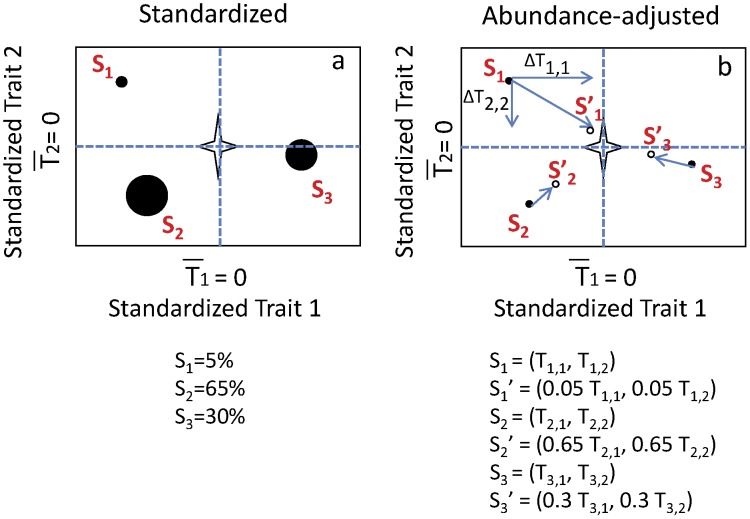
Illustration of abundance weighting procedure for FD and Hulls. Calculations are shown for a simplified community of three species with unequal abundances (abundance represented by the size of circles). Subscripts are for species i and trait j. Trait values for species are standardized to a mean of zero and standard deviation of one (Z-scores). Trait values for species are then multiplied by the proportional relative abundance (bound between zero and one), which results in a translation towards the origin, more so for rare species and less so for abundant species (see Appendix 1 for calculation). This modified distribution is then used for subsequent metric calculation. Weighing by the CV involves multiplying each standardized trait value by the CV (a positive value). This “stretches” trait axes with CV>1, effectively spreading species further apart along that axis, and “compresses” trait axes with CV<1, effectively crowding species closer together along that axis. We performed CV weighting prior to abundance weighting.

Two alternative abundance weightings were constructed for FD based on abundances from harvested clip strips. First, trait data for each species were weighted by individual species abundance (“FD_abun_”) prior to calculating multivariate distances. Since trait data were always scaled to center on zero (see below), and abundances were relative, ranging from 0–1, this weighting procedure moves rare species towards the centroid of the trait distribution (de-emphasizing their influence on trait diversity) while leaving abundant species comparatively unchanged (preserving their influence on trait diversity; Figure 1, Appendix 1). This adjustment alters the interpretation of the metric from a functional diversity metric, to an *effective* functional diversity metric based on abundance. For processes that scale positively with abundance, the metric will accentuate this linkage, while the metric will perform poorly for processes that scale independently with abundance. Abundance-weighting of convex hull volumes was done in an identical fashion, weighting trait values directly prior to calculating multivariate volume.

The second weighting approach for FD is similar in structure to Rao’s Q which weighs by the joint abundances of pairs of species (termed “FD_joint.abun_”; [18]). For this approach, the multivariate distances between species were weighted by the product of species relative abundances, prior to clustering into a functional trait dendrogram (i.e. the new distance between two species *d’* is related to the original distance, *d*, by: 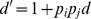, where *p_i_* and *p_j_* are the relative abundances of species *i* and *j*, respectively). We performed this calculation with and without unity and found no difference in prediction of ecosystem function. It is worth noting that recalculating dendrograms for each community has been previously proposed [45], and while this process differs from the original functional diversity index (based on the entire species pool), in practice the results are identical [49]. We also explored using abundance-adjustments using data from visually estimated percent cover subplots. Because adjustments using biomass data were often better predictors, and qualitatively similar to those with cover, we focus on the former.

In addition to abundance weighting, we investigated how variance-weighting of trait values alters functional diversity metrics. To perform this adjustment, after traits were standardized (mean zero standard deviation unity) we multiplied the trait value for each species by the coefficient of variation (CV) of the raw trait data. This process “stretches out” axes with a higher CV and “compresses” those with a lower CV, retains inter-species spacing, and emphasizes traits that have a higher degree of variation. We also performed this adjustment on Rao’s Q for comparative purposes.

### Analyses

We assessed correlations among the 19 diversity metrics. We used Spearman’s ρ throughout because several of the associations were nonlinear and some of the metrics were not normally distributed. To determine which metric(s) most accurately predicted ecosystem function, we ran analyses similar to those in previous examinations of this experiment [36], using a linear mixed-effects model with the diversity metric as the fixed effect, and plot within ring as a random effect (ambient CO_2_ in three of the six rings) across time to account for intra-plot dependencies through the long duration of the experiment. Analyses were run separately for each of the three functions of interest (aboveground biomass, light incident on the soil surface, and belowground biomass). We used Akaike weights to differentiate among models, with the best models scoring the highest Akaike weight, and other models scoring lower by comparison [50]. We carried out an additional analysis by combining multiple measures of functional diversity to test which set of metrics best predicted each function of interest. Two hundred twenty two combinations of metrics were assessed, out of many more possible ones; the selective set of combinations always included species richness, and then tested the addition of the dendrogram-based, functional richness, and functional dispersion measures. Linear regressions of selected relationships are provided for illustrative purposes. All models satisfied assumptions of homogeneity of variance and normality, and residuals were inspected for patterns and none were found.

## Results

### Associations among Predictors

**Figure 2:**
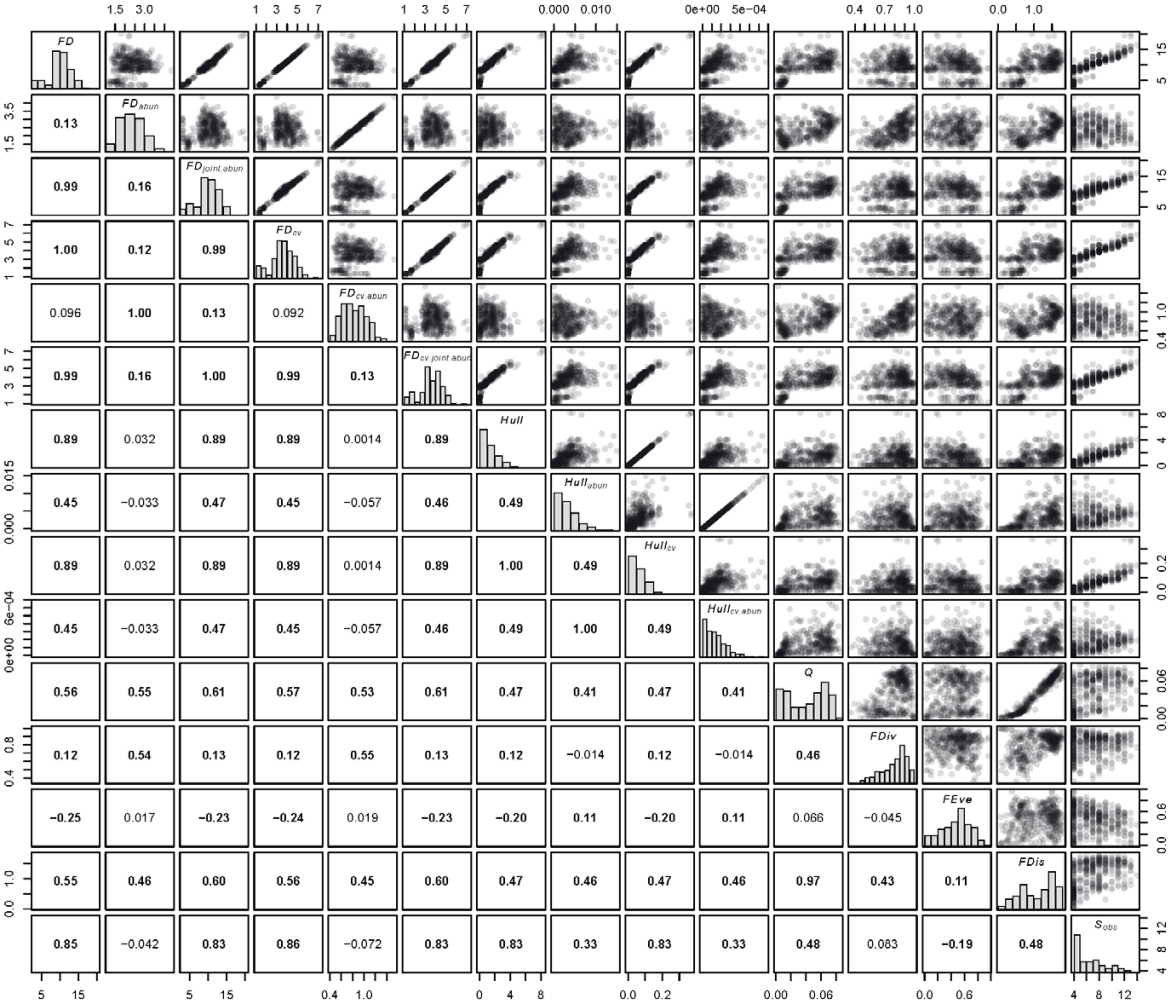
Associations among functional diversity metrics explored in this study. Shown below are bivariate plots (upper panels), distributions (diagonal), and Pearson’s ρ (lower panels, significant terms are in bold, P<0.05) for the 19 diversity metrics examined here.

All metrics except FDiv, FEve, FD_abun_, and FD_cv.abun_ tended to be highly correlated (average ρ >0.30) with other metrics, and all except FD_abun_ and FD_cv.abun_ were significantly correlated with species richness (whether planned or observed; Table 1, Figure 2). Simulation studies using randomly constructed communities have shown a correlation with species richness is not inherent to FDiv or FEve [24]. Associations were generally similar between species and functional group richness, and between planned and observed richness. Variations within Hull-based metrics were often highly correlated with one another regardless of adjustments (all ρ ≥0.49). Within FD metrics, those weighted by joint abundances (FD_joint.abun_, FD_cv.joint.abun_) were very highly correlated with unweighted FD (all ρ >0.95), while metrics weighted by trait abundances were not (Figure 2). CV-weighting had little effect on metric correlations, as the selected traits had similar levels of variation across the 16 species in this study.

### Predictions of Ecosystem Function

**Figure 3:**
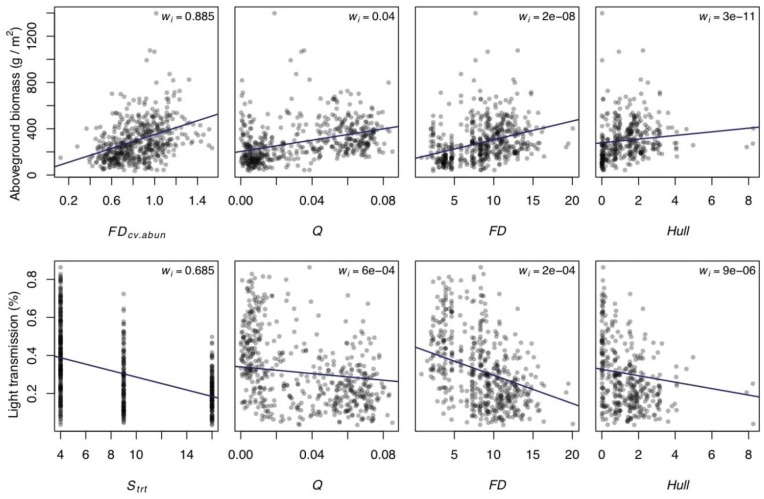
Illustrative bivariate plots for select functional diversity metrics and ecosystem function. Relationships between aboveground biomass (top row) or light (bottom row) with functional diversity metrics. Leftmost panels show the strongest predictors based on AIC, and selected representative metrics are shown to the right for comparison. Reproduced from Tables 1 and 2 are Akaike weights (w_i_), with larger weights indicating greater relative strength of evidence for that predictor.

Akaike weights suggested that the best single predictor was FD that was CV- and abundance-adjusted by traits, explaining approximately 36% of the variation in aboveground biomass (FD_cv.abun_, Table 2, Figure 3). Nonetheless, many diversity metrics explained similar amounts of variation in aboveground biomass (R^2^>0.3, Table 2), and all the best metrics and were positively and significantly associated with aboveground biomass. In particular, FD_cv.abun_, FD_cv_, Q, Q_cv_, FDis, and FDiv all performed similarly in terms of R^2^ in our analysis. However, Akaike weights gave virtually no support for any metrics other than FD_cv.abun_ (e.g. 10∶1 odds or worse for any of the other metrics, Table 1). Hereafter, we term FD_cv.abun_ as FD’ for simplicity. FD-based indices generally predicted aboveground biomass better than Hull-based indices, and all abundance-adjusted FD metrics performed better than their unadjusted counterparts. Other diversity metrics predicted aboveground biomass poorly by comparison. Qualitatively similar results were found for belowground biomass (Table 3).

**Table 2:** Results for linking functional diversity with aboveground biomass.

Metric	R^2^	ΔAIC	Akaike weight	Slope	P-value
FD_cv.abun_	0.362	0	0.906	61.71	<<0.001
FD_abun_	0.355	4.77	0.083	59.24	<<0.001
Q	0.387	10.25	0.005	61.56	<<0.001
Q_cv_	0.387	10.25	0.005	61.56	<<0.001
FDis	0.363	16.84	<0.001	56.63	<<0.001
FDiv	0.386	20.58	<0.001	46.97	<<0.001
FD_cv.oint.abun_	0.254	27.02	<0.001	52.30	<<0.001
FD_joint.abun_	0.254	27.62	<0.001	51.58	<<0.001
FD	0.235	29.70	<0.001	49.93	<<0.001
FD_cv_	0.240	29.91	<0.001	49.63	<<0.001
S_trt_	0.325	31.23	<0.001	48.88	<<0.001
FGR_trt_	0.331	32.27	<0.001	44.58	<<0.001
FGR_obs_	0.266	38.56	<0.001	33.96	<<0.001
S_obs_	0.270	40.99	<0.001	30.90	0.005
Hull	0.291	44.39	<0.001	19.11	0.066
Hull_ cv_	0.291	44.39	<0.001	19.11	0.066
FEve	0.312	46.01	<0.001	−10.38	0.249
Hull_ cv.abun_	0.313	47.17	<0.001	−0.54	0.956
Hull_ abun_	0.313	47.17	<0.001	−0.54	0.956

Summary of linear mixed-effects models for diversity metrics on aboveground biomass in the BioCON experiment. R^2^ are shown for observed versus predicted values. Comparisons are based on Akaike weights, with larger weights indicating greater relative strength of evidence for that predictor. Slopes are standardized and associated P-values are for significance of diversity metrics on aboveground biomass.

**Table 3:** Results for linking functional diversity with belowground biomass.

Metric	R^2^	ΔAIC	Akaike weight	Slope	P-value
FD_cv.abun_	0.330	0	0.861	−88.575	<<0.001
FD_abun_	0.321	3.68	0.137	−84.096	<<0.001
FGR_obs_	0.262	15.08	<0.001	74.296	<<0.001
FD_cv_	0.254	15.60	<0.001	74.608	<0.001
FD	0.253	15.62	<0.001	74.346	<0.001
FD_joint.abun_	0.251	17.13	<0.001	71.177	<0.001
FD_cv.joint.abun_	0.249	17.26	<0.001	70.749	<0.001
Hull	0.244	19.60	<0.001	60.603	0.001
Hull_cv_	0.244	19.60	<0.001	60.603	0.001
S_obs_	0.247	20.46	<0.001	63.238	0.001
FDiv	0.299	21.41	<0.001	−50.674	0.002
Q	0.318	22.06	<0.001	−58.774	0.001
Q_cv_	0.318	22.06	<0.001	−58.774	0.001
FGR_trt_	0.241	22.36	<0.001	58.066	0.006
S_trt_	0.241	24.94	<0.001	51.462	0.023
FDis	0.303	25.76	<0.001	−43.278	0.016
Hull_cv.abun_	0.252	27.95	<0.001	28.332	0.095
Hull_abun_	0.252	27.95	<0.001	28.332	0.095
FEve	0.266	28.65	<0.001	−23.250	0.140

Summary of linear mixed-effects models for diversity metrics on belowground biomass in the BioCON experiment. R^2^ are shown for observed versus predicted values. Comparisons are based on Akaike weights, with larger weights indicating greater relative strength of evidence for that predictor. Slopes are standardized and associated P-values are for significance of diversity metrics on belowground biomass.

Many diversity metrics also explained similar amounts of variation for light capture (Table 4). However, the best predictor for light capture was treatment species richness (S_trt_, Table 4, Figure 3), with increases in the diversity metric associated with increased light capture. Treatment functional group richness (FGR_trt_) performed similarly, while other diversity metrics predicted light capture poorly by comparison on the basis of model fit (Table 4).

**Table 4:** Results for linking functional diversity with light capture.

Metric	R^2^	ΔAIC	Akaike weight	Slope	P-value
S_trt_	0.260	0	0.921	−0.082	<<0.001
FGR_trt_	0.263	4.98	0.076	−0.069	<<0.001
FD_cv.joint.abun_	0.242	13.83	0.001	−0.052	<<0.001
FD_joint.abun_	0.241	13.89	0.001	−0.052	<<0.001
FD_cv_	0.234	16.14	<0.001	−0.048	<<0.001
FD	0.234	16.91	<0.001	−0.046	<0.001
S	0.222	21.14	<0.001	−0.038	0.002
FDis	0.284	24.23	<0.001	−0.022	0.018
Q	0.289	24.58	<0.001	−0.022	0.023
Q_cv_	0.289	24.58	<0.001	−0.022	0.023
Hull	0.235	25.19	<0.001	−0.020	0.047
Hull_ cv_	0.235	25.19	<0.001	−0.020	0.047
FGR	0.239	25.92	<0.001	−0.020	0.082
FD_cv.abun_	0.215	26.98	<0.001	0.011	0.164
FD_abun_	0.218	27.11	<0.001	0.011	0.182
FDiv	0.228	27.92	<0.001	0.008	0.333
Hull_abun_	0.246	28.59	<0.001	−0.002	0.784
Hull_cv.abun_	0.246	28.59	<0.001	−0.002	0.784
FEve	0.245	28.62	<0.001	−0.004	0.589

Summary of linear mixed-effects models for diversity metrics on light incident on the soil surface in the BioCON experiment. R^2^ are shown for observed versus predicted values. Comparisons are based on Akaike weights, with larger weights indicating greater relative strength of evidence for that predictor. Slopes are standardized and associated P-values are for significance of diversity metrics on light not captured by the canopy.

## Discussion

Several criteria have been proposed for the selection of a suitable index of functional diversity: (1) the metric should measure what it is intended to describe, (2) the metric should be uncorrelated with other metrics, and (3) the metric should conform to certain expectations and mathematical properties (usually more important for functional richness indices). Parallel to the above criteria is the acknowledgement that most, if not all, metrics represent one attribute or another of functional diversity to varying degrees [1], [24], [51], [52]. Indeed, our overall findings suggest that for this system functional richness (estimated by FD_cv.abun_) was statistically the best predictor, although other metrics for functional evenness (Q) and functional divergence (FDiv) also predicted aboveground biomass fairly well. The choice of the three traits focused on in this study did not bias the results, as a re-analysis with additional traits demonstrated (Table S1). In this re-analysis, it was necessary to exclude the Hulls metrics to still analyze the four species communities, as Hulls requires fewer traits than species.

We additionally ran supplemental analyses examining all possible models with one to six linear combinations of metrics to explore the hypothesis that the best models overall for aboveground biomass would incorporate all three aspects of functional diversity (Table S2). Best models invariably included FD’, as well as species richness, functional group richness, Rao’s Q, and then a combination two terms (Hull or Hull_abun_ combined with FDis or FEve; all four combinations). Mouillot et al. (2011) found that some combination of FDiv, FEve, and Hulls (termed FRic in that publication) consistently predicted decomposition, productivity, and nutrient cycling, using several different analytical approaches. Indeed, FDiv appeared the most predictive single metric of that set, with the highest function when abundant species were quite different from one another. Similar combinations were not similarly predictive in our system.

One reason for these differences could be related to the size of trait space sampled in this experiment. Namely, that the multidimensional size of trait space sampled in our experiment (Minnesota prairie species) may have been demonstrably smaller than that in Mouillot et al (2011) (mid European hay meadow). In any generic community, as the total volume of trait-space occupied declines, the ratio of the multidimensional surface to volume increases. Thus, the potential explanatory power of richness measurements (the surface) should increase as the total trait volume sampled declines, a hypothesis that deserves testing. Because all species in our system are adapted to a fairly harsh environment, they represent a restricted subset of trait combinations already. Thus, we might expect functional richness estimates (like FD’) to be larger in their relative explanatory power in simpler communities than metrics describing the filling of trait space.

What is the abundance- and trait variation-weighted version of FD (FD’) really measuring? The strong association commonly reported between unadjusted FD and species richness [1] was eliminated with abundance-adjustments. FD' was most highly correlated with Rao’s Q (ρ = 0.38), suggesting that like Q it might represent multiple attributes of functional diversity simultaneously. This could be considered a strength or a weakness, depending on whether your priority is centered on disaggregating different components of diversity, or on developing a useful summary variable. Conceptually, FD’ measures the functional richness within the community, discounting species that are rare, aggregating species that are similar, and increasing contributions from traits that are inherently more variable. Thus, only species or species-groups that are functionally distinct based on trait-abundance combinations contribute in a meaningful way to the index. This also means that rare species that are very different from each other have similar (minor) influences on ecosystem function, which may or may not enhance prediction depending on the degree to which abundance translates to function. This is neither desirable nor undesirable for a metric, but merely accentuates abundant and different species. We suggest interpreting FD’ as a measure of effective functional richness rather than absolute or potential functional richness, which unadjusted richness measures more faithfully describe. A more comprehensive assessment of the behavior of FD’ is underway (Flynn et al. *unpublished data*). Nonetheless, this smoothing over of the trait variation within a community by FD’ appears to strengthen the linkage between functional diversity and aboveground biomass.

Several other generalizations emerged from our analysis of above- and belowground biomass. First, our finding that abundance weighting greatly improved the association between functional diversity and biomass production, is strong support for Grime’s “mass ratio” hypothesis. We acknowledge that the scaling of function with abundance is not always the case [53], but our results demonstrate a strong mass ratio effect for biomass production. Second, although variance weighting generally improved the predictive power of diversity metrics over their unweighted counterparts, this improvement was much more subtle because the CV’s for our traits were very similar. Nonetheless, heterogeneous variation among traits is common and we feel should be incorporated into any comprehensive measure of functional diversity. Third, dendrogram-based diversity metrics greatly outperformed Hulls, suggesting that the functional associations among all species within a community are more predictive of ecosystem function than associations among species with extreme trait values (but see [51]). Fourth, joint-abundance weighting (e.g. Q, FD_joint.abun_) performed much more poorly than single-abundance weighting on species trait values (e.g. FD’). Weightings based on the product of abundances of two species emphasize evenness more than trait distinctiveness, which did not enhance predictive ability in our system.

**Figure 4:**
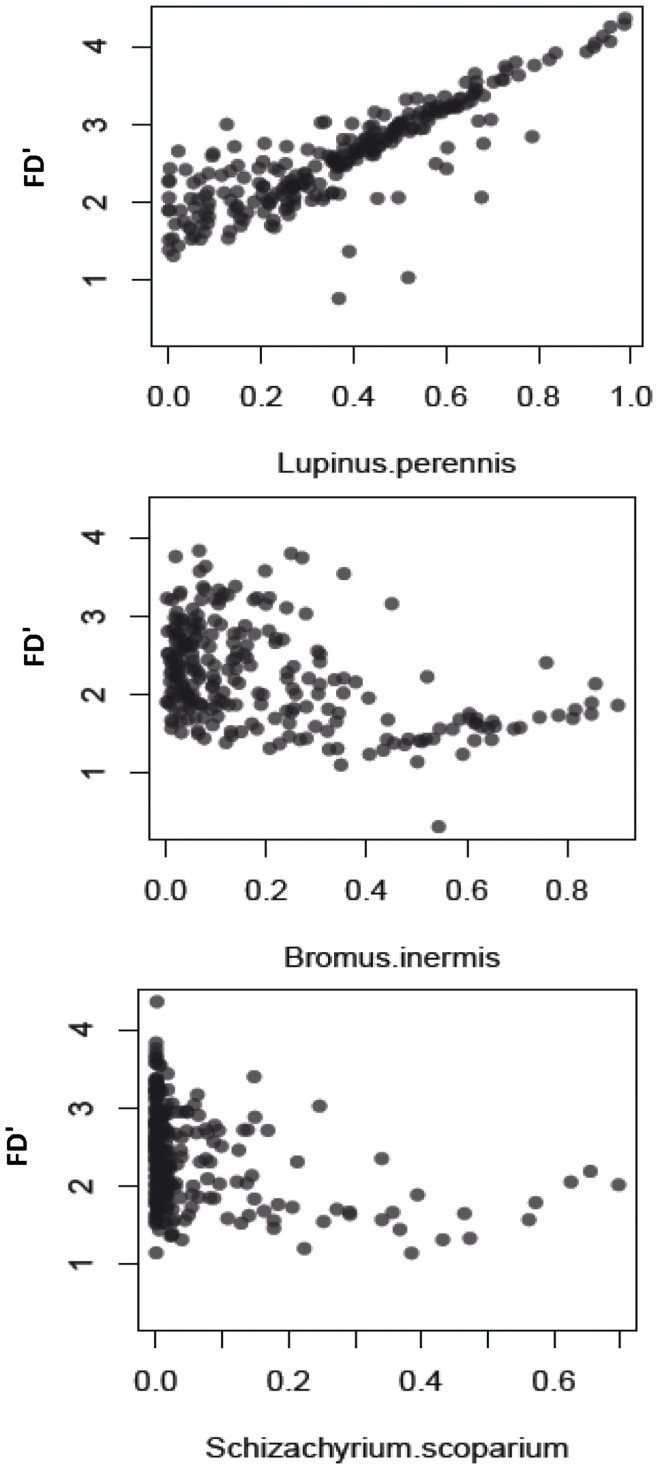
Associations between FD’ and species abundances. Shown are associations between FD’ and three example species: *Lupinus perennis* (N-fixer), *Bromus inermis* (C3 grass) and *Schizachyrium scoparium* (C4 grass).

**Figure 5:**
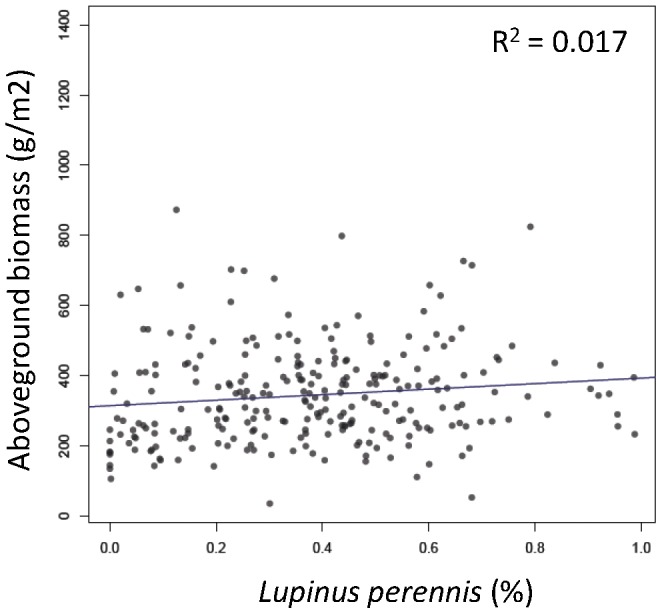
Association between *Lupinus perennis* and aboveground biomass. Linear association between the relative abundance of *Lupinus perennis* and aboveground biomass.

We ran preliminary analyses to explore the behavior of FD’ more fully. FD’ was not highly correlated with the abundance of any species in this system except the legume *Lupinus perennis* (Figure 4), with greater FD’ values as *Lupinus* became more dominant. The association with the legume however was not inherent to the metric. For FD’, as a species becomes increasingly abundant within the community, the value of the metric increasingly represents the multidimensional distance between that species and the centroid of the community. Thus, the value of FD’ could increase or decrease as a species came to dominate depending on whether that target species was different from, or similar to, the other species in the community (e.g. increasing for *Lupinus* and decreasing for *Bromus*, respectively, Figure 4). As the abundance of the target species continues to increase, FD’ declines to zero because the individual abundance-adjusted distances approach zero either from low abundances (for rare species) or a low distance to the centroid (for the dominant species). Thus, FD’ is maximized when several species that are very different co-dominate, similar in concept to a one-dimensional approximation of FDiv [24]. Thus, although the relative abundance of *Lupinus* alone was not a strong predictor of total aboveground biomass (R^2^ = 0.02, Figure 5), plots were especially productive when *Lupinus* coexisted with a diverse assemblage of species that were different from itself (e.g. C4 grasses). This is a general result that has been reported for this and other studies but never synthesized into one metric (e.g. [27]). This association may prove general if tested in other real (not simulated) systems.

Our results revealed some important differences between analyses on randomly assembled communities versus real communities. Schleuter et al. (2010) found that dendrogram-based measures of functional diversity (termed FRD there) were uncorrelated with other diversity indices, and several other studies have found that Rao’s Q is not strongly associated with species richness [1], [13]. Both of these contrast with our results, and suggest that the assembly process in real communities can cause associations to emerge between functional diversity metrics which are not mathematically predetermined.

The notable mismatch we found between results for the three ecosystem properties was not expected given the direct association between aboveground biomass, belowground biomass, and light interception. Both aboveground and belowground biomass were well predicted by only a few traits related to production of photosynthate (SLA, leaf N) and its relative allocation above- and belowground (RMF), which were selected *a priori* based on previous research. The addition of seed mass, height, and specific root length strengthened the performance of abundance-weighted FD, although the CV-weighted version became slightly less predictive (Table S1). On the other hand, light capture appears to be a much more complex process, and was best predicted by species richness alone. This discrepancy is likely explained in that light interception is a function of not only the total aboveground biomass, but also the geometric configuration of the canopy. This might be expected for a community such as ours that includes species with generally vertical foliage (monocots) as well as more heterogeneous and horizontal foliage (dicots). None of the traits we examined were related to canopy architecture. Strong relationships with richness rather than species-level trait-based metrics might also be expected if there is significant plasticity at the individual level of traits, in this case leaf and stem deployment. Thus, each additional species appeared to “fill in” the canopy, resulting in species richness predicting light interception best. Incorporation of additional traits such as leaf angle and plasticity in leaf and stem deployment may enhance our ability to predict light capture from functional traits. More generally, the discrepancy between response variables in this study stresses that no combination of traits is likely to be universally applicable to the study of all ecosystem functions, even those that are closely related such as biomass accumulation and light interception.

Predicting ecosystem function requires incorporating contributions from several interacting sources, including the regional climate, biogeochemical attributes of the habitat, and characteristics from the biota [54]. Our analyses suggest that the biotic contribution to predicting ecosystem function is larger when trait-based measures of functional diversity are utilized that include contributions from all species within the community, and that incorporate heterogeneous variation in species abundances and in trait variation. However, this result holds only for the ecosystem function for which traits were specifically selected (aboveground biomass), and surprisingly, not for a closely related function (light capture). The finding of an association between a functional diversity metric, and a particular function of interest, does not by itself establish an association between functional diversity (the concept) and ecosystem function, nor does it invalidate the value of alternative metrics for describing functional diversity. Different functional diversity metrics highlight different aspects of functional diversity. Species richness highlights the aspect of uniqueness, where every species is valued equally irrespective of traits, while functional evenness (e.g. FEve, [24]) highlights the evenness of spread for physical traits within the community. Different circumstances (i.e. functions of interest) will likely favor some metrics more than others in terms of predictability. We feel that continued study on which underlying attributes of functional diversity matter for which function of interest would greatly advance the field of ecology.

### Conclusion

Interest in continuous measurements of functional diversity has grown substantially in recent years, with an ever-growing number of metrics available for researchers to use. These metrics perform in different ways, and capture different aspects of biological communities [55]. For researchers interested in understanding the consequences of biodiversity loss, until recently there have been no direct comparison of these predictors with experimental data [51]. Here, we have provided another such comparison, and tested several established metrics against hybrid metrics that combine approaches that have shown prior success. We found that even though our new metric based on abundance- and variance- adjusted dendrograms outperformed other metrics for aboveground biomass, several existing metrics performed similarly. Each of these metrics represent valid and different attributes of functional diversity, the combination of which is likely to better predict ecosystem function. The choice of which traits to include in any measure of functional diversity remains crucial and should be tailored to the ecosystem process of interest. Moving towards consensus in how to assess functional diversity will aid in the work to both understand the processes regulating community assembly and the consequences of biodiversity for ecosystem processes.

## Supporting Information

Table S1 **Summary of model comparison results for when using six traits.**
(DOCX)

Table S2 **Summary of model comparison for multi-metric assessment of predicting aboveground biomass.**
(DOCX)

Appendix S1 **Calculations for abundance weighting FD and Hulls.**
(DOCX)
