# Supplementary material for: Testing the Link between Functional Diversity and Ecosystem Functioning in a Minnesota Grassland Experiment
Source: PLoS One. 2012 Dec 31;7(12):e52821. doi: 10.1371/journal.pone.0052821 (PMC3534119; doi:10.1371/journal.pone.0052821)
Supplement: Table S1 — Summary of model comparison results for when using six traits. (DOCX) [file pone.0052821.s001.docx]

**Table S1:** Summary of model comparison results for when using the six traits SLA, N, root:shoot ratio, seed mass, height, and specific root length, and excluding convex hull volumes. For all metrics, slopes of responses are highly significant.

| Response variable | Metric | R^2^ | ∆AIC | Akaike weight | Slope |
| --- | --- | --- | --- | --- | --- |
| Aboveground biomass | FD_abun_ | 0.321 | 0 | 0.59 | 50.49 |
|  | FD_cv_ | 0.263 | 1.83 | 0.24 | 63.52 |
|  | FD_cv.joint.abun_ | 0.269 | 3.48 | 0.10 | 62.22 |
|  | FD | 0.242 | 5.63 | 0.04 | 60.56 |
|  | FD_joint.abun_ | 0.251 | 7.61 | 0.01 | 58.76 |
|  | FD_cv.abun_ | 0.302 | 8.20 | 0.01 | 48.09 |
|  | Q | 0.267 | 9.34 | 0.01 | 51.23 |
|  | Q_cv_ | 0.267 | 9.34 | 0.01 | 51.23 |
|  | FDis | 0.262 | 16.63 | 0.00 | 44.18 |
|  | S_trt_ | 0.271 | 18.25 | 0.00 | 51.75 |
|  | FGR_trt_ | 0.277 | 20.64 | 0.00 | 45.70 |
|  | FGR_obs_ | 0.260 | 23.32 | 0.00 | 40.83 |
|  | S_obs_ | 0.265 | 28.33 | 0.00 | 35.31 |
|  | FDiv | 0.322 | 28.66 | 0.00 | 24.59 |
|  | FEve | 0.299 | 37.77 | 0.00 | -6.03 |
| Light transmission | FGR_trt_ | 0.501 | 0 | 0.97 | -0.09 |
|  | S_trt_ | 0.502 | 7.37 | 0.02 | -0.09 |
|  | FD_cv_ | 0.495 | 12.25 | 0.00 | -0.08 |
|  | FD_cv.joint.abun_ | 0.499 | 13.53 | 0.00 | -0.07 |
|  | FD | 0.487 | 14.76 | 0.00 | -0.07 |
|  | FD_joint.abun_ | 0.491 | 17.12 | 0.00 | -0.07 |
|  | FGR_obs_ | 0.483 | 27.32 | 0.00 | -0.05 |
|  | S_obs_ | 0.493 | 27.83 | 0.00 | -0.05 |
|  | FDiv | 0.518 | 32.30 | 0.00 | 0.02 |
|  | Q | 0.503 | 36.54 | 0.00 | -0.01 |
|  | Q_cv_ | 0.503 | 36.54 | 0.00 | -0.01 |
|  | FDis | 0.503 | 36.82 | 0.00 | -0.01 |
|  | FD_abun_ | 0.515 | 37.11 | 0.00 | 0.01 |
|  | FD_cv.abun_ | 0.513 | 38.06 | 0.00 | 0.01 |
|  | FEve | 0.511 | 38.64 | 0.00 | 0.00 |
| Belowground biomass | FD_abun_ | 0.300 | 0 | 0.87 | -77.70 |
|  | FGR_obs_ | 0.265 | 3.88 | 0.12 | 85.97 |
|  | FGR_trt_ | 0.251 | 10.65 | 0.00 | 77.85 |
|  | FD_cv.abun_ | 0.294 | 11.93 | 0.00 | -63.03 |
|  | S_obs_ | 0.267 | 12.68 | 0.00 | 72.24 |
|  | S_trt_ | 0.262 | 17.98 | 0.00 | 63.53 |
|  | FD | 0.270 | 18.81 | 0.00 | 56.86 |
|  | FDiv | 0.293 | 19.31 | 0.00 | -41.19 |
|  | FD_joint.abun_ | 0.269 | 20.85 | 0.00 | 49.27 |
|  | FDis | 0.296 | 23.95 | 0.00 | -32.96 |
|  | Q | 0.294 | 24.13 | 0.00 | -32.98 |
|  | Q_cv_ | 0.294 | 24.13 | 0.00 | -32.98 |
|  | FD_cv_ | 0.271 | 24.16 | 0.00 | 34.19 |
|  | FD_cv.joint.abun_ | 0.272 | 25.28 | 0.00 | 26.18 |
|  | FEve | 0.282 | 25.36 | 0.00 | -21.33 |
