# Supplementary material for: Testing the Link between Functional Diversity and Ecosystem Functioning in a Minnesota Grassland Experiment
Source: PLoS One. 2012 Dec 31;7(12):e52821. doi: 10.1371/journal.pone.0052821 (PMC3534119; doi:10.1371/journal.pone.0052821)
Supplement: Table S2 — Summary of model comparison for multi-metric assessment of predicting aboveground biomass. (DOCX) [file pone.0052821.s002.docx]

**Table S2:** Summary of model comparison for multi-metric assessment of functional diversity metrics as predictors of aboveground biomass. A selected set of the many possible combinations shown below. See main text for explanation of metrics.

| Rank | Metric | R^2^ | AIC | Akaike weight | Intercept |
| --- | --- | --- | --- | --- | --- |
| 1 | S_trt_+FGR_trt_+FD_abun_._cv_+Hull_abun_+Q+FDis | 0.240 | 0 | 0.089 | 281.42 |
| 5 | S_trt_+FGR_trt_+ FD_abun.cv_ +Hull+Q+FDis | 0.245 | 0.42 | 0.072 | 284.77 |
| 9 | S_trt_+FGR_trt_+ FD_abun.cv_ +Hull+Q+FEve | 0.261 | 3.09 | 0.019 | 284.96 |
| 13 | S_trt_+FGR_trt_+ FD_abun.cv_ +Hull_abun_+Q+FEve | 0.259 | 3.14 | 0.019 | 283.81 |
| 129 | S_trt_+ FD_abun.cv_ +FDis | 0.243 | 19.20 | <0.001 | 286.39 |
| 130 | S_trt_+ FD_abun.cv_ +Q | 0.246 | 19.54 | <0.001 | 286.90 |
| 136 | S_trt_+FGR_trt_+FD+Hull_abun_+Q_cv_+FEve | 0.199 | 20.74 | <0.001 | 275.05 |
| 148 | S_trt_+ FD_abun.cv_ +Hull_abun_ | 0.246 | 21.40 | <0.001 | 286.96 |
| 150 | S_trt_+FGR_trt_+FD_abun_+Hull_cv.abun_+Q+FEve | 0.209 | 21.50 | <0.001 | 275.97 |
| 154 | S_trt_+ FD_abun_ +FDis | 0.238 | 25.25 | <0.001 | 286.39 |
| 179 | FD_abun.cv_ | 0.298 | 49.13 | <0.001 | 302.25 |
| 180 | S_trt_+FD_abun_+Hull | 0.176 | 49.15 | <0.001 | 249.97 |
| 190 | FD_abun_ | 0.289 | 53.91 | <0.001 | 301.97 |
| 191 | Q_cv_ | 0.214 | 59.45 | <0.001 | 288.19 |
| 193 | S_trt_+FD_cv.abun_+Hull_cv.abun_ | 0.166 | 60.63 | <0.001 | 274.96 |
| 203 | FDis | 0.202 | 65.93 | <0.001 | 286.94 |
| 204 | S_trt_+FD+FEve | 0.179 | 66.13 | <0.001 | 279.06 |
| 208 | FDiv | 0.317 | 69.81 | <0.001 | 296.81 |
| 209 | S_trt_+FD | 0.176 | 70.62 | <0.001 | 279.03 |
| 212 | FD_cv.abun_ | 0.160 | 75.98 | <0.001 | 281.91 |
| 213 | FD_joint.abun_ | 0.163 | 76.59 | <0.001 | 282.44 |
| 214 | FD | 0.154 | 78.68 | <0.001 | 282.22 |
| 215 | FD_cv_ | 0.157 | 78.87 | <0.001 | 281.89 |
| 216 | S_trt_ | 0.218 | 80.60 | <0.001 | 281.84 |
| 217 | FGR_trt_ | 0.231 | 81.11 | <0.001 | 281.40 |
| 218 | Hull | 0.240 | 93.69 | <0.001 | 300.13 |
| 220 | FEve | 0.272 | 95.09 | <0.001 | 295.65 |
| 221 | Hull_abun_ | 0.271 | 96.48 | <0.001 | 296.35 |
