# Supplementary material for: Testing the Link between Functional Diversity and Ecosystem Functioning in a Minnesota Grassland Experiment
Source: PLoS One. 2012 Dec 31;7(12):e52821. doi: 10.1371/journal.pone.0052821 (PMC3534119; doi:10.1371/journal.pone.0052821)
Supplement: Appendix S1 — Calculations for abundance weighting FD and Hulls. (DOCX) [file pone.0052821.s003.docx]

**Appendix S1:** Calculations for abundance weighting by species abundances (FD_abun_, and related metrics). Shown below are the calculations for FD_abun_ (see Figure 1 in text for reference) for a simplified system of three species. Species i (S_i_) is has traits j (T_i,j_) that are first standardized to a mean of zero and a standard deviation of one. The coordinates of species i in trait space is shown in equation 1 in the example. The abundance-adjusted coordinates (S’_i_) is simply the proportional abundance (0 to 1) multiplied by each standardized trait value (equation 2). The delta ($\Delta T_{i,j}$) is one minus the proportion to endure rare species move more and abundant species move less. In the example in Figure 1, species 1 moves further towards the centroid because it is rare, species 2 moves little because it is abundant, and species 3 is intermediate. The calculations explicitly showing the steps from equation 1 to equation 2 are shown in equation 3-6.

1. $S_{1}=\left( T_{1,1}, T_{1,2} \right)$
2. $S_{1}'=\left( {p_{1}T}_{1,1}, {p_{1}T}_{1,2} \right)$
3. $S_{1}'=\left( T_{1,1}+\Delta T_{1,1}, T_{1,2}+\Delta T_{1,2} \right)$
4. $\Delta T_{1,1}=\left( 1-p_{1} \right)\left( \bar{T}_{1}-T_{1,1} \right)={p_{1}T}_{1,1}-T_{1,1}$
5. $\Delta T_{1,2}=\left( 1-p_{1} \right)\left( \bar{T}_{2}-T_{1,2} \right)={p_{1}T}_{1,2}-T_{1,2}$
6. $S_{1}^{'}=\left( T_{1,1}+{p_{1}T}_{1,1}-T_{1,1}, T_{1,2}+{p_{1}T}_{1,2}-T_{1,2} \right)=\left( {p_{1}T}_{1,1}, {p_{1}T}_{1,2} \right)$
